# Ceramic-Based Piezoelectric Material for Energy Harvesting Using Hybrid Excitation

**DOI:** 10.3390/ma14195816

**Published:** 2021-10-05

**Authors:** Bartłomiej Ambrożkiewicz, Zbigniew Czyż, Paweł Karpiński, Paweł Stączek, Grzegorz Litak, Łukasz Grabowski

**Affiliations:** 1 Department of Automation, Faculty of Mechanical Engineering, Lublin University of Technology, Nadbystrzycka 36, 20-618 Lublin, Poland; p.staczek@pollub.pl (P.S.); g.litak@pollub.pl (G.L.); 2 Aeronautics Faculty, Military University of Aviation, 08-521 Dęblin, Poland; z.czyz@law.mil.pl; 3 Faculty of Mechanical Engineering, Lublin University of Technology, Nadbystrzycka 36, 20-618 Lublin, Poland; pawel.karpinski@pollub.edu.pl (P.K.); l.grabowski@pollub.pl (Ł.G.)

**Keywords:** hybrid energy harvesting system (HEHS), Macro Fiber Composite (MFC), cross-section design, vortex-induced vibration (VIV), galloping

## Abstract

This paper analyzes the energy efficiency of a Micro Fiber Composite (MFC) piezoelectric system. It is based on a smart Lead Zirconate Titanate material that consists of a monolithic PZT (piezoelectric ceramic) wafer, which is a ceramic-based piezoelectric material. An experimental test rig consisting of a wind tunnel and a developed measurement system was used to conduct the experiment. The developed test rig allowed changing the air velocity around the tested bluff body and the frequency of forced vibrations as well as recording the output voltage signal and linear acceleration of the tested object. The mechanical vibrations and the air flow were used to find the optimal performance of the piezoelectric energy harvesting system. The performance of the proposed piezoelectric wind energy harvester was tested for the same design, but of different masses. The geometry of the hybrid bluff body is a combination of cuboid and cylindrical shapes. The results of testing five bluff bodies for a range of wind tunnel air flow velocities from 4 to 15 m/s with additional vibration excitation frequencies from 0 to 10 Hz are presented. The conducted tests revealed the areas of the highest voltage output under specific excitation conditions that enable supplying low-power sensors with harvested energy.

## 1. Introduction

Energy Harvesting (EH) is a very wide field of science that consists of energy conversion from the external environment [1,2], and its concepts gain popularity in real applications [3,4]. Most miniaturized systems are quite good independent power supplies for low-power electronic devices mounted in hardly accessible environments such as open water [5], other planets [6] or mines [7]. Energy harvesting perfectly fits in the modern philosophy of “green technology” and uses the natural sources of energy like solar [8], wind [9], or energy harvested from sea waves [10], in a friendly way.

One of the challenges in the development of EH systems is to maximize their efficiency and adaptability to external excitation. In order to obtain the maximum output power, such systems are designed to operate in their resonance frequency, however, in the real environment there is no single frequency acting on the system. Unfortunately, different frequency components acting on the system are observed at the spectra as other peaks or super- or sub-harmonics [11,12]. Another challenge is to find the optimal operating conditions coming from multiple excitations and control them to achieve the longest possible lifetime of the EH system. The analysis of hybrid excitation has attracted the attention of several researchers [13,14,15,16,17]. Following this trend, an original concept of the hybrid energy harvesting system based on the piezoelectric effect was proposed. For the analysis of system performance, wind energy, and mechanical vibrations were applied to find the most desirable conditions of excitation (air flow velocity and excitation frequency) and the optimal design (few masses were considered).

The considered excitation in form of air flow is so called flow-induced vibrations and can lead to the following instabilities: vortex-induced vibrations (VIV) [18], flutters in airfoils [19], galloping in prismatic structures [20], and meta-structures analysis, by which one bluff body affects the excitation of another one [21]. Following the research of Wang et al. [22] and Yangs et al. [23,24] on the mixed-cross section of bluff bodies, by which circular cross-section leads to VIV and a square cross-section leads to the galloping phenomena. In his work, he studied the mixed cross-section with the number of square corners circumcircled in the circle. Referring to that design, the authors of this paper propose the modified geometry of a bluff body, whose cross-section changes over its length from a circle to a square.

The vortex-shedding phenomena behind the bluff body was studied in other research. Akaydin [25] analyzed short, flexible piezoelectric cantilever beams placed inside turbulent boundary layers and wakes of circular cylinders at high Reynolds numbers.

The model that directly predicts levels of energy harvested from large oscillations of an object subjected to wake galloping was presented in [26]. Its validation was performed by experiments in a suction-type open circuit wind tunnel. The research object was a set of two cuboidal cylinders placed in the wake of a circular cylinder in the test section.

Moreover, certain customized shapes of a bluff body are studied in an analysis of piezoelectric energy harvesting systems. The paper by Seyed-Aghazadeh et al. [27] investigates the L-shaped beam in which structural stiffness led to nonlinear effects. The proposed design is an alternative to the classical cantilever beam. The introduced modification enhanced the power density of the EH system.

Another work worth mentioning on the wind energy harvester was presented in the paper of He et al. [28]. In order to increase system efficiency, the interaction between vortex-induced vibration and galloping, which is dependent on bluff body geometries, was proposed.

Another issue is the transverse galloping phenomena studied by Barrero-Gil et al. [29]. They refer to fluid-elastic instability that occurs if the critical wind velocity is exceeded. Oscillations are then excited in the transverse direction to the wind. Such oscillations have a negative influence on the system performance because they lead to critical oscillations. In the research, it was proved that efficiency of energy harvesting can be significantly improved by using an active rotation of the galloping body.

This work considers a mixed design of the bluff body (different shapes of cross-section) and its different masses. As it was mentioned, the square-shaped body is sensitive to the vortex induced vibrations, while the circular-shaped body has the tendency to the galloping effect. Countermeasures are often taken to avoid mentioned self-excited vibrations occurring in building structures, such as chimneys, towers, high voltage lines, or bridge decks. However, in the case of aviation or energy harvesting solutions, both effects can have a desirable and positive impact on the dynamical response [30]. Increased vibrations during the self-excited vibrations tend to excite the system with high amplitudes, what has a direct effect on the power output. The additional study on optimal performance was coupled with hybrid excitation in the form of variable air flow velocity in the wind tunnel, and an application of external excitation of mechanical vibrations coming from the shaker. Additionally, the initial analysis in the form of flow terms calculation was conducted and the output voltage obtained with the piezo-element, and an acceleration of tip mass were calculated for different flow velocities and excitations.

The remainder of this paper is as follows: Section 2 describes the design of the energy harvester and test rig, Section 3 presents the experimental procedure with a specification of experimental cases, Section 4 presents the data processing and discusses the voltage and acceleration results, and the last section summarizes the conducted research.

## 2. Design of the Hybrid Piezoelectric Energy Harvester (HPEH) and Test Stand

**Figure 1:**
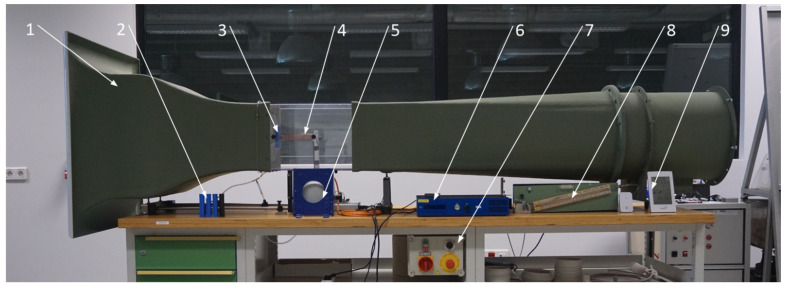
General view of the experimental test rig. 1, HM 170 open wind tunnel (GUNT Hamburg); 2, set of the studied bluff bodies; 3, bluff body in the test section; 4, cantilever beam with the piezo-element; 5, vibration generator; 6, digital amplifier; 7, control panel of the wind tunnel; 8, measurement of air-flow velocity; 9, weather station.

**Figure 2:**
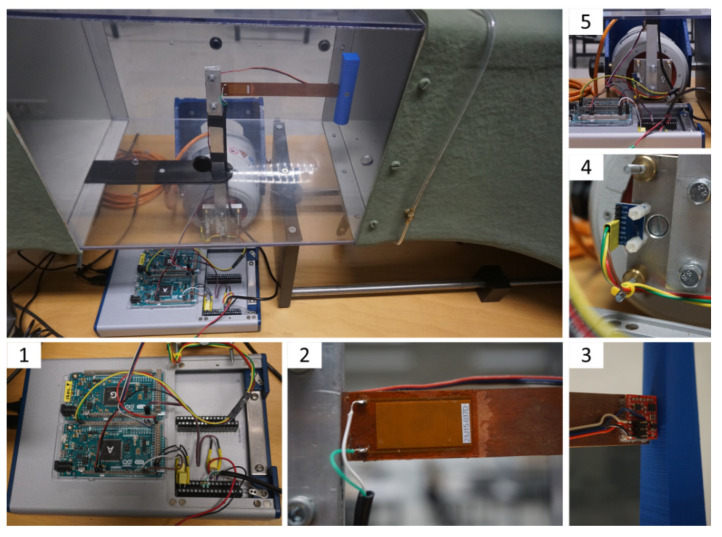
View of the test section and measuring system. 1, USB-6341 data acquisition board with Arduino Due; 2, piezo-element; 3, accelerometer at the tip of cantilever beam–the bluff body (its cross-section is changing smoothly from circular to square downwards); 4, accelerometer on the vibration generator; 5, vibration generator with the vertical support structure.

In this chapter, the test rig and bluff-body models used in the experiment are described. The wind tunnel with the marked elements of the measuring system is depicted in Figure 1. The piezoelectric harvester (Figure 2) consists of the cantilever beam, piezoelectric element, bluff body, and support structure. The cantilever beam is made of copper alloy and its dimensions are 200 × 20 mm^2^ (length × width), but 20 mm of its length was used for its support and the same length for the placement of the bluff body. The beam was screwed to the support structure. The alignment of the bluff body and the cantilever beam allows oscillations in the horizontal axis. Such an orientation of the beam was applied due to the induced vortices behind the bluff body, generating the pressure differences on both sides of the beam. The research object was placed in the test section in the subsonic wind tunnel with an open loop. The cross-section of the test section is 300 × 300 mm^2^, square-shaped, and the research object was placed centrally. In the conducted experiment, the flow velocity over the object was controlled by changing the rotational velocity of the fan installed at the wind tunnel outlet. In order to obtain a stable wind flow, a honeycomb structure is installed at its inlet.

A variety of materials can be used as energy harvesters, for example, ZnO (Zinc Oxide) nanowires placed directly on titanium foil [31]. In our research, the Macro Fiber Composite™ (MFC) P1 type from Smart Material was applied as a piezoelectric material. The applied piezoelectric material consists of the following layers: the mid-layer is a monolithic PZT wafer, which is a ceramic-based piezoelectric material, and the outer layers are an interdigitated electrode pattern on polyimide film [32]. Piezoceramic fibers are placed in an epoxy matrix, which prevents crack propagation, and a patterned alignment of the electrodes ensures higher electromechanical efficiency. Such an innovation improves both resilience and elasticity in comparison with monolithic ceramics [33]. After applying the voltage, it acts as an actuator and is able to bend the material to excite or damp vibrations. If no voltage is applied, it acts as a very sensitive strain gauge detecting vibrations and deformations. This is why the MFC is a very efficient material for energy harvesting from external excitation. It works as an elongator and utilizes the d33 effect for actuation. It can elongate up to 1800 ppm if operated at the maximum voltage rate from −500 V to +1500 V [34]. The selected technical features of the studied MFC piezo-element are provided in Table 1.

For beam excitation, the TIRA S513 vibration generator (TIRA GmbH, Schalkau, Germany) was applied. The electrodynamic generator was mounted on the special support structure that enables operation under different angles, both horizontally and vertically. Nominal displacement was achieved by using a pneumatic load compensation, even for heavy test loads. The support structure was equipped with vibration isolators capable of minimizing the vibration transfer to the base surface. The generator was cooled by a free fan and the air passed through the filter assembly [35]. The selected technical features of the used vibration generator are in Table 2.

The excitation sinusoidal signal was amplified with the TIRA DA 200 digital amplifier (TIRA GmbH, Schalkau, Germany). The device can cooperate with permanent magnet-driven shakers and is characterized by a high signal-to-noise ratio and a low distortion factor. The selected technical parameters of the device are provided in Table 3.

**Figure 3:**
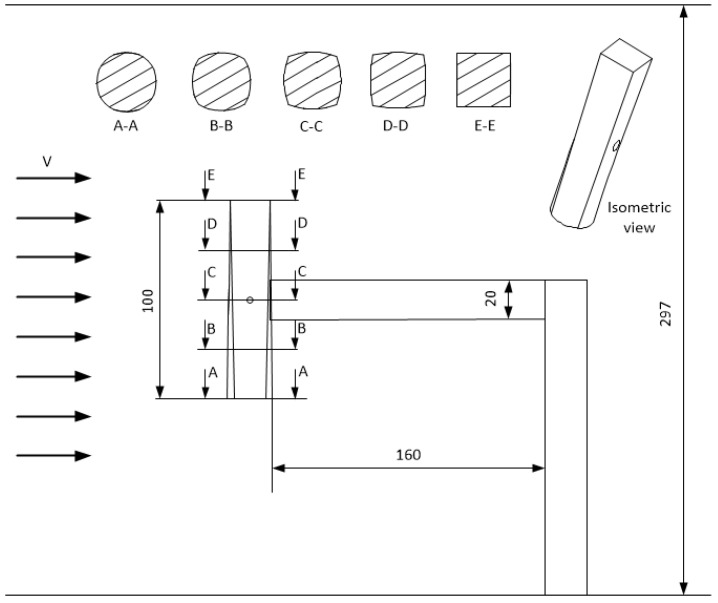
Bluff body geometry and the selected cross-sections.

The research object (Figure 3) was a bluff body, which is a hybrid of a cylinder and a cuboid. The geometries of the bluff body were designed in the Solidworks 2018 CAD software and then created with a 3D printer Ender 5 Plus (Creality, Lublin, Poland) using the FDM (Fused Deposition Modeling) method. The shape of the surface was smoothly changed along the length of the bluff body from a square to a circle using the spline function. It should be noted that the cross-section areas of the square and circle at the ends are equal to 400 mm^2^. The selected cross-sections of the bluff-body and its assembly with the support structure are depicted in Figure 3. The motivation and the novelty for mixed cross-section of the bluff body was the possibility to observe different types of self-excited vibrations (i.e., vortex induced vibrations and the galloping effect). The length of each bluff body was 100 mm. Each of the research objects had a pocket on its sidewall to be connected to the cantilever beam.

The motivation for choosing the square-circle design was based on the conclusions in the research of Wang et al. [22]. The authors of that research claim that a cuboid bluff body leads to galloping, while a circular shape causes VIV (vortex induced vibrations). Such phenomena can occur for a high Reynolds number only. However, the authors did not consider the combination of both shapes, which could bring together the advantages of both VIV and galloping to enhance energy scavenging. In our research, we have decided to combine square and circular shapes to observe an unknown type of vibration. The motivation for conducting the experiment is the evaluation of system efficiency, correlated with voltage output. Moreover, hybrid excitation was applied in the form of a sinusoidal signal and the mass of each bluff body was changed. Five bluff bodies of different masses were studied in the experiment. The masses of the samples were as follows: 15.7 g, 21.0 g, 30.4 g, 37.8 g, and 45.6 g. These different masses of the bluff body were obtained by the change of infill percentage from 20% to 100% during the 3D printing process with an increment of 20%.

**Figure 4:**
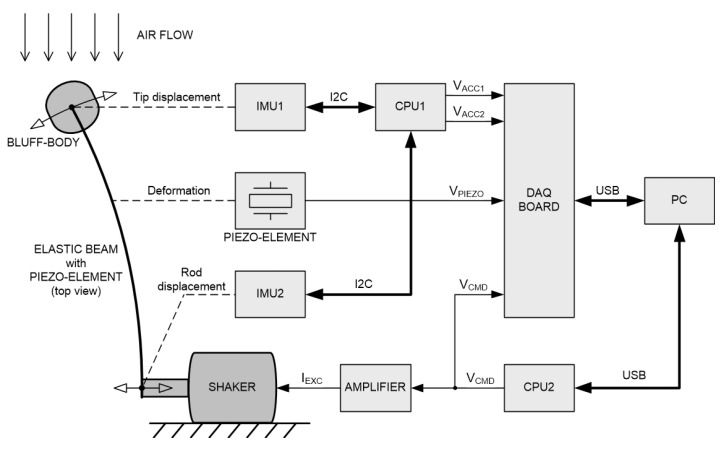
Diagram of the measuring system.

The sine wave of the desired displacement for the shaker rod (V_CMD_, voltage commands in Figure 4) was generated by a 32-bit microcontroller (CPU2, central processing unit in Figure 4, Arduino Due, Torino, Italy). The amplitude and frequency of this signal were changed automatically in accordance with the preset sequence set by the software in the PC. The digital amplifier (AMPLIFIER (TIRA GmbH, Schalkau, Germany)) fed the shaker winding with the current I_EXC_(t) (excitation current) proportional to the signal V_CMD_(t). Additionally, the V_CMD_(t) wave was recorded along with the other signals.

The linear accelerations of the bluff body and shaker rod (pusher) were measured using MEMS (microelectromechanical system) accelerometers (SparkFun, Boulder, CO, USA) type MMA8452Q (up to −/+8 g range, 12-bit resolution, 800 Hz output data rate)-blocks IMU1 and IMU2 (inertial measurement unit) in Figure 4. The acceleration measurement results were sent to a 32-bit microcontroller (CPU1, Arduino Due, Torino, Italy)) via the I2C digital communication bus and then immediately converted to voltage analog signals V_ACC1_ and V_ACC2_ (acceleration voltage), feeding the inputs of a National Instruments USB-6341 data acquisition board (DAQ BOARD, National Instruments, Austin, TX, USA). All of the measured signals were synchronously digitally recorded with a frequency of 800 Hz using the FlexLogger software (2021 R1) from National Instruments.

## 3. Experimental Procedure and Data Processing

The aim of the research was the evaluation of voltage efficiency of the proposed energy harvesting system under the hybrid excitation induced by variable wind-flow velocity and external sinusoidal excitation. Five different bluff bodies of variable mass were studied. The sinusoidal excitation was induced by a vibration generator connected to the support structure. The wind-flow velocity was controlled in the test section by changing the rotational velocity of the fan. In the experiment, the generated voltage by the piezo-element and the acceleration of tip mass were recorded. The values of excitation parameters are in Table 4. The system stabilized between each experimental case within 30 s. The voltage and acceleration were registered within the same time, and the experiment consisted of 390 cases.

The Reynolds number for the studied bluff bodies was changing in the range from 5000 to 19,000. According to the theoretical-experimental studies for small values of the Reynolds number (below 50), laminar or steady flows are observed. The air-flow studies of the cylindrical bluff body for low values of the Reynolds number and a consideration of vortex shedding were presented in [36]. The mentioned value (i.e., vortex shedding) occurs in alternating circulation, which is called a von Kármán vortex street. For high Reynolds numbers (over 10^5^), the flow is unstable and turbulent [37]. However, the air flow behind the bluff body is not typically formed in a von Kármán vortex street predicted for an infinitely long cylinder. Another feature that characterizes the studied phenomena is the Strouhal number. It is associated with flow oscillations due to inertial forces relative to changes in velocity due to convective acceleration of the flow field. For small values of the Strouhal number, oscillations are damped; for high ones, they have a dominating effect on the flow, and for the intermediate values, vortex shedding occurs. For a certain range of Reynolds numbers, a vortex street is created. The value of the Strouhal number for a fixed bluff body is calculated from the following equation [38]:

(1) $$St=\frac{fL}{U}\;\;\;$$

where *f* is frequency of vortex shedding, *L* is characteristic length, and *U* is flow velocity.

For the considered cases, the Strouhal number was in the range from around 0.004 to 0.019. These values were calculated only for the cases of bluff-body vibration due to air flow. This number was not calculated for the forced vibration cases.

The experiment was followed by the signal processing of the obtained time-series for each case calculated in the Matlab 2020a software. For the results of the output voltage generated with the piezo-element with a resistive load of 1 MΩ, the Root Mean Square (RMS) was calculated as the basic measure of system efficiency by the specific flow velocity and harmonic excitation. Similarly to the voltage calculation, the acceleration of tip mass was obtained from the following formula [39]:

(2) $$a_{RMS}=\sqrt{\frac{1}{N}\sum_{n=1}^{N}{\left|a_{n}\right|}^{2}},$$

where *n* is the number of samples in the time-series.

## 4. Discussion

**Figure 5:**
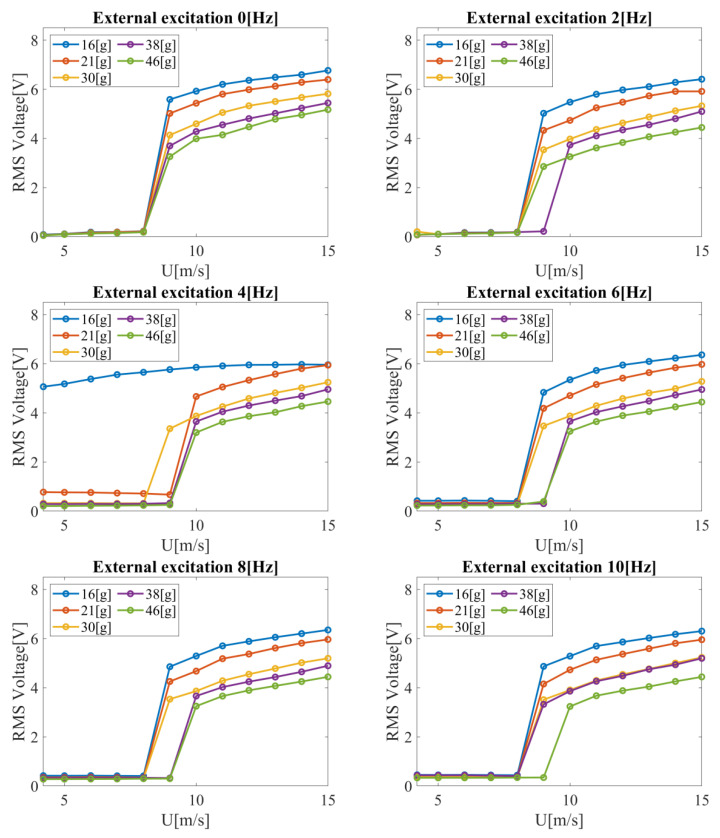
RMS voltage on the piezoelectric element as a function of air flow velocity for the considered bluff body masses.

The voltage as a function of airflow velocity for the considered values of mechanical excitation was plotted and the obtained results are summarized in Figure 5. The results can be divided into two ranges. The former is for low flow velocities up to 8 or 9 m/s, and the latter for higher velocity values. In the first range, extinguished vibrations with small amplitudes were observed (even with the active shaker). Regarding energy recovery, the developed system is clearly dedicated to the second range (above 8 or 9 m/s), where the vibration amplitude intensively increases. This leads to high voltage values obtained on the piezoelectric element. Considering the results obtained in the version without the external excitation, for the smallest mass of the tested bluff body (i.e., *m*_1_= 15.7 g), the maximum RMS voltage value equal to 6.766 V was obtained at the flow velocity *u* = 15 m/s. The RMS voltage value decreased with an increase in mass. For the next tested mass (i.e., *m*_2_ = 21.0 g), the RMS voltage equal to 6.395 V was obtained and is lower by 5.5% in comparison to mass m_1_. The further increase of the bluff body mass generated a decrease in RMS voltage by 14.0%, 19.5%, and 23.6%, respectively. It can be concluded that increasing bluff body mass has a damping effect on the system.

Decreasing air velocity from *u* = 15 m/s to *u* = 9 m/s also decreases RMS voltage. For mass *m*_1_, this is a decrease of as much as 17.5%. A larger decrease in vibration is observed for the other masses. Hence, the RMS voltage values decreased by 21.5%, 28.9%, 32.1%, and as much as 37.0%, respectively. Both mass and flow velocity have a significant effect on system performance. The decreased air flow velocity increases the difference of the generated voltage with respect to the analyzed maximum velocity. For a flow velocity of *u* = 14 m/s and a bluff body mass of *m*_1_ = 15.7 g, a maximum RMS value of 6.593 V was obtained. With an increase in mass, as before, a decrease in the RMS voltage value was observed. For the next investigated mass, *m*_2_ = 21.0 g, RMS voltage equal to 6.283 V was obtained, which is lower by 4.7% in relation to the mass *m*_1_. A further increase of the bluff body mass generated a decrease in RMS voltage by 14.1%, 20.6%, and 24.8%, respectively. For *u* = 9 m/s, the maximum voltage drop for mass *m_5_* relative to *m*_1_ was as high as 41.7%.

After a series of wind tunnel tests, the experiments were continued for the case with external excitation. After the analysis of the results, it was found that the excitation of the system in the given frequency range negatively influences the voltage generated by the piezoelectric material. In each of the considered cases, there was a decrease in RMS voltage due to the activation of the vibration generator.

Among the results obtained, only for *m*_1_ and *f* = 4 Hz did the bluff body vibrate with a large amplitude over the entire range of airflow velocities. This is evident in Figure 5 and can be explained by the fact that this particular frequency is the frequency close to the resonance of the system. For the other considered configurations, the bluff body did not vibrate so intensively at velocities below 8 or 9 m/s. In the other cases, there was also no significant effect of the frequency of the forced vibration on the RMS voltage generated. Activating the vibration generator with a frequency in the range from 2 to 10 Hz resulted in a decrease in RMS voltage by a minimum of 11.4%, but a maximum of as much as 19.7%. The average RMS voltage drop for all measurement points with the vibration generator was equal to 14.6%.

**Figure 6:**
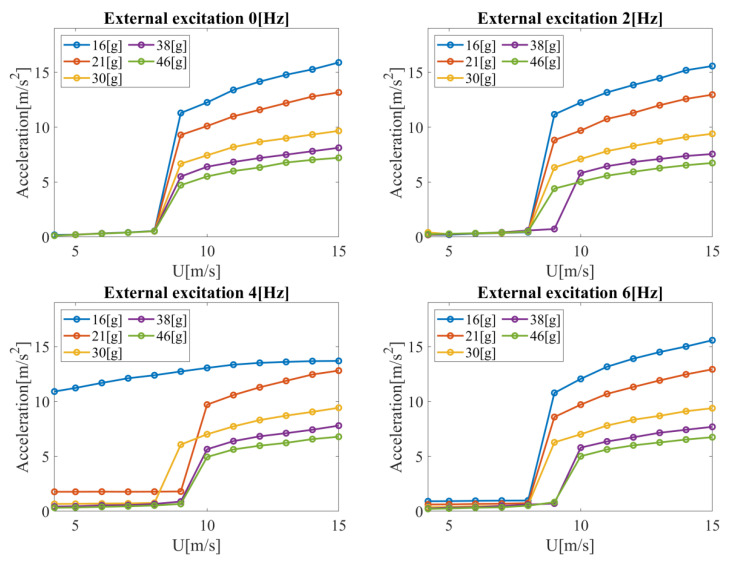

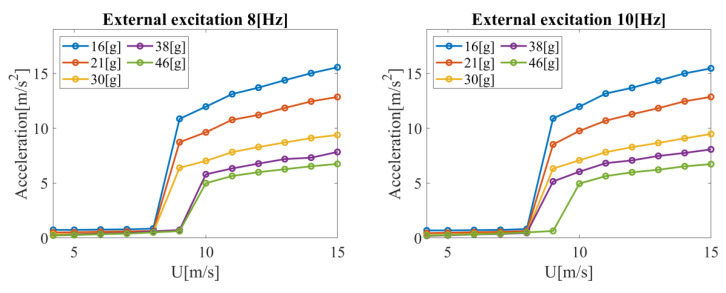
Acceleration of the end of the cantilever beam with the piezoelectric element as a function of air velocity for the considered bluff body masses.

The observed high amplitude vibrations for a frequency of 4 Hz and a mass of 15.7 g and the obtained amplitudes for the case without air flow leads to the following conclusion: the high efficiency of the piezoelectric element can be achieved by applying considerably smaller external excitation. It is considered to perform experiments in the frequency range from 2 to 4 Hz (Figure 6). For this range, resonant frequencies occur and an efficient operation of the piezoelectric element over the whole range of investigated velocities can be expected.

A similar analysis was performed for the acceleration signals of the end of the cantilever beam for the considered bluff body masses (Figure 6). The nature of qualitative changes for these signals is analogous to the voltage signals discussed above. The differences are in the quantitative aspect. Acceleration for all considered mass configurations, frequencies, and airflow velocities did not exceed 16 m/s^2^. In the case without external excitation, the acceleration value increased with increasing mass. An increase in the external excitation frequency did not cause a significant change in the acceleration value. The highest difference occurred at a frequency of 4 Hz and a mass of 15.7 g. This case was close to the resonance frequency of the tested system. Therefore, non-zero acceleration values (from 10.9 to 13.7 m/s^2^) were observed for the entire range of airflow velocities. The acceleration value was initially close to zero in all other cases considered (the beam remained steady). At a certain velocity limit, there was a sharp increase in acceleration due to the excitation of the beam. This limit value depended on the frequency of external excitation and was equal to about 8–9 m/s. A further increase in velocity resulted in an approximately linear increase in acceleration.

**Figure 7:**
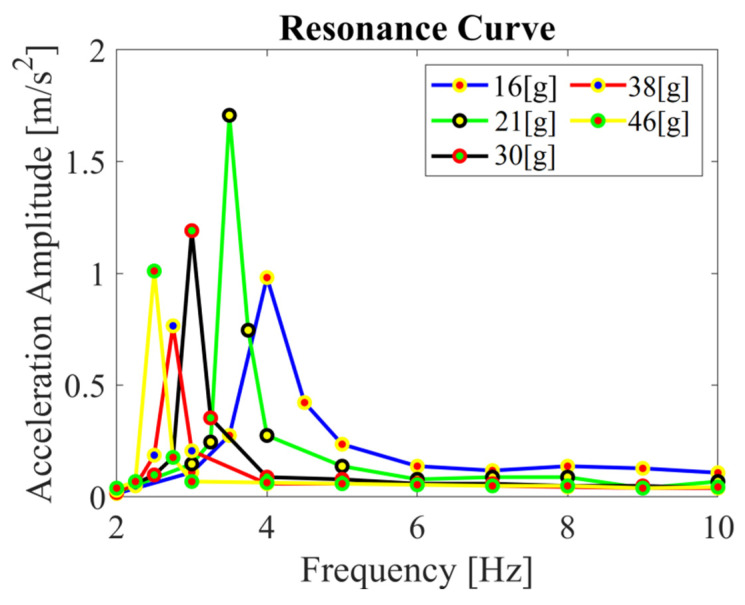
Acceleration amplitude as a function of excitation frequency (FFT analysis) for the considered bluff body masses for the case without airflow.

Considering the efficiency of voltage generation by the tested system, it is important to determine the value of the resonant frequency for which the vibrations have the largest amplitude. For this frequency, it is expected that even a small periodic forcing force can cause oscillations of a significant amplitude. The force in the considered system comes from the periodically flowing vortices from the bluff body. The benefit of operating the system at resonant frequencies is demonstrated by the case described above for the lowest mass at *f* = 4 Hz which is resonant frequency for this configuration. Future work will investigate cases for the resonant frequencies obtained. The acceleration amplitude in the frequency domain for the case without external excitation was also analyzed (Figure 7). It was observed that the resonant frequency decreased with increasing the bluff body mass. The highest acceleration amplitude (1.71 m/s^2^) was obtained for the bluff body mass of 21.0 g and the lowest (0.77 m/s^2^) for the mass of 37.8 g. These points corresponded to frequencies of 3.5 Hz and 2.75 Hz, respectively.

**Figure 8:**
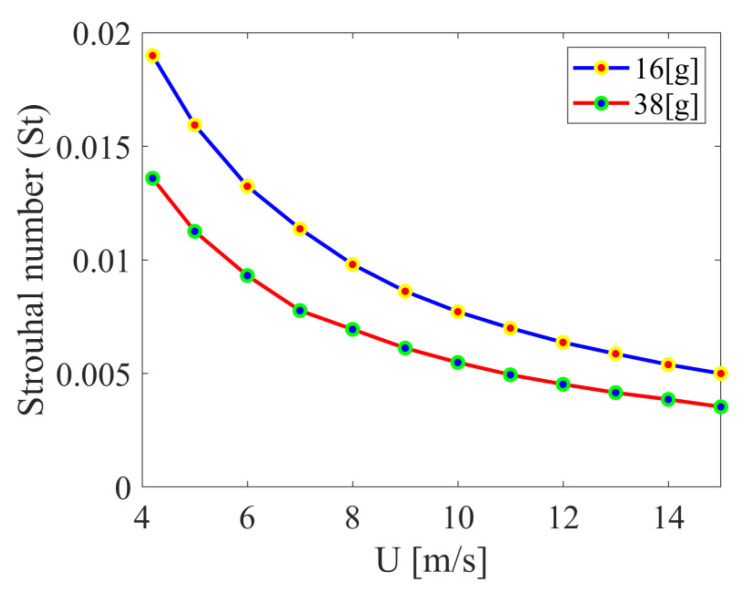
Strouhal number *St* as a function of air flow velocity for the two bluff body masses for the case without external excitation. The natural frequency of the vibrating bluff body was used in Equation (1) as the specific frequency *f*.

Besides defining the Reynolds number range, the Strouhal number as a function of flow velocity for two bluff body masses without external excitation was determined. The obtained values are displayed in Figure 8. Generally, as the air flow velocity increased, the Strouhal number decreased. The obtained value of the Strouhal number varied from 0.005 to 0.019 for the smallest sample mass (15.7 g). For the larger sample mass (37.8 g), lower values of the Strouhal number were obtained for the whole range of air flow velocities (from 0.004 to 0.014); this is related to the increase in inertial forces. The low Strouhal number values are evidence of vibration absorption by the fast-flowing fluid.

## 5. Conclusions

This paper analyzes the energy efficiency of an MFC piezoelectric system. It is a smart material that consists of a monolithic PZT wafer, which is a ceramic-based piezo-electric material. The investigated system consisted of a bluff body in the form of a unique geometry that is a combination of a square and circular cross-section. The bluff body was mounted vertically in the test section in the wind tunnel, perpendicular to the horizontal beam with the piezo-element. This position of the test body enabled the use of vortices generated by the bluff body to induce vibrations of the system. Moreover, the study included additional external excitation at a fixed frequency using a mechanical vibration generator. The developed test rig allowed for the recording of a voltage signal generated by the piezoelectric element and an acceleration signal from an accelerometer placed on a cantilever beam near the bluff body. The tests were carried out for five objects differing in mass.

Voltage and acceleration were recorded for certain fixed values of flow velocity in the wind tunnel and set values of excitation frequency. The main objective of this work was to analyze the energy efficiency of the investigated energy harvester in the form of a bluff body connected to a piezo-element placed on a cantilever beam.

For the configuration without excitation (*f* = 0 Hz), high values of generated voltage in the velocity range above 8 m/s were observed. The maximum RMS voltage values obtained during the tests did not exceed 7 V. Increasing the mass had a negative effect on the results obtained in this range. Rapid vibration damping was observed for all tested bluff bodies at 8 m/s.

The shape used, which is a hybrid combining square and circular cross-sections, is an example of a solution between galloping and VIV phenomena. The tested object is susceptible to vibrations without external excitation at higher velocities (above 8 m/s) but with strong damping properties at lower ones.

The correct selection of external excitation frequencies and bluff body masses enabled high voltage values over the entire air velocity range. This means that it is possible to extract energy from the environment using the proposed geometry of the bluff body even at low air velocity values.

The tests were conducted for relatively low Strouhal numbers leading mainly to the galloping movement. This was due to the small dimension of the bluff body and the shape of the geometry which is hardly susceptible to high vibration frequencies. Its hybrid nature enhances vibration damping in the considered flow velocity range. Notably, the larger *St* realized for a fairly small mass can coincidentally switch on the VIV and higher voltage output with smaller *U* in the presence of mechanical excitation in the resonance zone

The mechanical vibrations generated by the shaker were of a constant amplitude. Until now, the effects of air flow velocity in the wind tunnel and vibration frequency on the efficiency of the system were investigated. An additional outcome is the finding of the resonance frequency of a specific bluff body coupled with the support structure. Moreover, the wind velocity providing the transition between stable state and oscillations for bluff bodies was found experimentally at *U* = 8 m/s or 9 m/s, depending on the external excitation and mass. Mentioned observations allow focus on the oscillation solution of the bluff body on the cantilever beam to obtain the highest possible value of the output voltage.

No vibration of the piezo-element beam was observed for low velocities. According to previous studies [22], the cylinder-type shape tends to have a VIV phenomenon that occurs in the low air velocity range. It follows that, to obtain beam vibration for low velocities and thus energy recovery in this state by the energy harvester, it is necessary to increase the percentage of cylindrical cross section in the considered bluff body.

Further work will focus on an analysis of different amplitudes of forced vibrations at frequencies below 4 Hz, since in this range the resonance frequencies for the investigated bluff bodies were obtained. According to the obtained results, the high system efficiency in the whole range of flow velocities was obtained only for the measurement point for *f* = 4 Hz and the smallest mass of the bluff body. The planned tests will be followed by specifying the values of parameters for which the system operates efficiently over the whole range of the tested velocity.

## Figures and Tables

**Table 1:** Technical parameters of MFC P1 piezo-element.

Parameter	Value/Description
Dimensions	Active: 28 × 14 mm^2^; Overall: 38 × 20 mm^2^
Max. blocking force	146 N (±10%)
Free strain	1160 ppm (±10%)
Max. operating voltage	−500 V to +1500 V
Max. operating frequency	Harvester < 1 MHz
Typical lifetime	Harvester: 10E + 10 cycles (<600 ppm)
Typical thickness	300 µm, 12 mil
Typical capacitance	1.9 nF (±20%)

**Table 2:** Technical parameters of TIRA S513 vibration generator.

Parameter	Value/Description
Rated peak force	100 N
Frequency range	2–7000 Hz
Max. rated travel	13 mm
Max. velocity	1.5 m/s
Max. acceleration	45 g
Rated current	5.5 A
Nominal impedance	4 Ω
Suspension stiffness	8 N/mm
Effective moving mass	0.23 kg
Main resonance frequency	>6500 Hz
Weight with a trunnion	10 kg
Armature	60 mm

**Table 3:** Technical parameters of TIRA DA 200 digital amplifier.

Parameter	Value/Description
Max. output power	200 VA
Frequency range	1.5–22,000 Hz
Max. voltage RMS	30 V
Max. current RMS	10 A
Signal input voltage RMS	7 V
Distortion	<0.1%
Signal-to-noise ratio	>90 dB

**Table 4:** Experiment design.

Parameter	Value
Mass	15.7 g; 21.0 g; 30.4 g; 37.8 g; 45.6 g
Frequency	f = {0, 2, 4, 6, 8, 10} Hz
Air velocity	U = {0, 4, 5, 6, 7, 8, 9, 10, 11, 12, 13, 14, 15} m/s

## Data Availability

Data available on request due to privacy restrictions.
